# Heterogeneity of *KRAS* Mutation Status in Rectal Cancer

**DOI:** 10.1371/journal.pone.0153278

**Published:** 2016-04-11

**Authors:** Peter Jo, Alexander König, Markus Schirmer, Julia Kitz, Lena-Christin Conradi, Azadeh Azizian, Markus Bernhardt, Hendrik A. Wolff, Marian Grade, Michael Ghadimi, Philipp Ströbel, Hans-Ulrich Schildhaus, Jochen Gaedcke

**Affiliations:** 1 Department of General, Visceral and Pediatric Surgery, University Medical Center Goettingen, Goettingen, Germany; 2 Department of Gastroenterology and Gastrointestinal Oncology, University Medical Center Goettingen, Goettingen, Germany; 3 Department of Clinical Pharmacology, University Medical Center Goettingen, Goettingen, Germany; 4 Department of Pathology, University Medical Center Goettingen, Goettingen, Germany; 5 Radiotherapy and Radiology Munich, Munich, Germany; National Cancer Center, JAPAN

## Abstract

**Introduction:**

Anti-EGFR targeted therapy is of increasing importance in advanced colorectal cancer and prior *KRAS* mutation testing is mandatory for therapy. However, at which occasions this should be performed is still under debate. We aimed to assess in patients with locally advanced rectal cancer whether there is intra-specimen KRAS heterogeneity prior to and upon preoperative chemoradiotherapy (CRT), and if there are any changes in *KRAS* mutation status due to this intervention.

**Materials and Methods:**

*KRAS* mutation status analyses were performed in 199 tumor samples from 47 patients with rectal cancer. To evaluate the heterogeneity between different tumor areas within the same tumor prior to preoperative CRT, 114 biopsies from 34 patients (mean 3 biopsies per patient) were analyzed (pre-therapeutic intratumoral heterogeneity). For the assessment of heterogeneity after CRT residual tumor tissue (85 samples) from 12 patients (mean 4.2 tissue samples per patient) were analyzed (post-therapeutic intratumoral heterogeneity) and assessment of heterogeneity before and after CRT was evaluated in corresponding patient samples (interventional heterogeneity). Primer extension method (SNaPshot™) was used for initial *KRAS* mutation status testing for Codon 12, 13, 61, and 146. Discordant results by this method were reevaluated by using the FDA-approved *KRAS* Pyro Kit 24, V1 and the *RAS* Extension Pyro Kit 24, V1 Kit (therascreen® *KRAS* test).

**Results:**

For 20 (43%) out of the 47 patients, a *KRAS* mutation was detected. With 12 out of 20, the majority of these mutations affected codon 35. We did not obtained evidence that CRT results in changes of the *KRAS* mutation pattern. In addition, no intratumoral heterogeneity in the *KRAS* mutational status could be proven. This was true for both the biopsies prior to CRT and the resection specimens thereafter. The discrepancy observed in some samples when using the SNaPshot™ assay was due to insufficient sensitivity of this technique upon massive tumor regression by CRT as application of the therascreen® *KRAS* test revealed concordant results.

**Conclusion:**

Our results indicate that the *KRAS* mutation status at the primary tumor site of rectal cancer is homogenous. Its assessment for therapeutic decisions is feasible in pre-therapeutic biopsies as well as in post-therapeutic resected specimens. The amount of viable tumor cells seems to be an important determinant for assay sensitivity and should thus be considered for selection of the analytical method.

## Introduction

Targeted therapy against the epidermal growth factor receptor (EGFR) represents a well-accepted and effective treatment strategy in metastatic colorectal cancer associated with an increased response rate and prolonged patient survival [1, 2]. In its oncogenic function *EGFR* controls crucial cellular functions such as differentiation, proliferation and survival making it a worthwhile target for anti-cancer therapy [3]. However, inhibition of this central signaling molecule in colorectal cancer is only promising when intracellular effectors downstream of *EGFR* are not altered by activating mutations. One of the major intracellular effectors translating *EGFR* signals represent the small GTPases *KRAS* controlling vital intracellular signaling cascades such as the Mitogen-Activated-Protein- (MAP-) Kinase signaling pathway. Genomic mutation in the *KRAS* gene locus results in a continuous activation of the MAPKinase pathway independent from extracellular factors and from the *EGFR*. Not surprisingly and due to continuous stimulation of the oncogenic signaling pathways by mutatively activated *KRAS*, inhibition of *EGFR* had no beneficial or even adverse effects in colorectal cancer patients harboring a *KRAS* mutation. Consequently, patients with mutated *KRAS* are excluded from anti-*EGFR* therapy and determination of the *KRAS* mutation status is required prior to intended therapy.

In general, *KRAS* mutation status is assessed by analyzing primary tumor tissue. However, due to intratumoral heterogeneity [4–6] *KRAS* mutation heterogeneity within different areas of the tumor might be a potential concern as recently reported at least for selected patients [7, 8]. A phenomenom that is not only restricted to colorectal cancer as shown by Queirós et al. [9]. Differences in the *KRAS* mutation status between primary tumor and distant metastases [7, 10]. as well as tumor stage dependency [11] has also been reported. There is certainly a high concordance between primary tumor and metastases as recently revealed by a meta-analysis from Han and colleagues [12]. However, it should also be taken into consideration that the mutation testing methodology utilized may be source of reported discrepancies as recently pointed out by Sherwood et al. in lung cancer [13].

In contrast to colon cancer preoperative chemoradiotherapy (CRT) is the treatment strategy of choice in patients with locally advanced rectal cancer. However, there is no agreement about the timing of the determination of the *KRAS* mutation status. Additionally, prospective results concerning concordance of the *KRAS* mutation status in pre- and post-therapeutic tumor samples (referred to as intertumoral heterogeneity) are rare and of conflicting nature [14–16]. Furthermore, there are no results demonstrating reproducibility of *KRAS* testing in the primary biopsy as well as in the resected specimen within the same tumor tissue (referred to as intratumoral heterogeneity). We therefore aimed to assess the *KRAS* status in multiple pre-therapeutic biopsies as well as in resected tumor specimens. Goal was to compare the mutation status before and after chemoradiotherapy in rectal cancer and to clarify if mutation differences in treatment naive tumors occur at all.

## Materials and Methods

### Patients and Treatment

Patients included in this analysis were treated at the Departments of General, Visceral and Pediatric Surgery and Radiotherapy and Radiation Oncology, University Medical Center Goettingen, and were enrolled or treated according to the trial guidelines of the CAO/ARO/AIO-94 [17] or CAO/ARO/AIO-04 [18] (EudraCT-Number 2006-002385-20—NCT00349076) of the German Rectal Cancer Study Group. All patients were followed-up according to the trial protocols and gave written informed consent either from the patients or their legal representatives. This study conformed with the ethical principles of the Declaration of Helsinki (Seoul, 2008) and was approved by the University of Goettingen Ethics Committee in Goettingen, Germany (application number 20/9/95, 9/8/08).

### Ascertainment of Pre- and Post-therapeutic Tumor Biopsies

Tumor biopsies were collected prior to preoperative CRT during the diagnostic procedures. Using typical biopsy forceps biopsies yielded the size of a pinhead. After surgery residual tumor was taken from the resected specimens. Due to tumor regression the entire tumor region was embedded and was later assessed by the pathologist. The mutation status was assessed in formalin-fixed-paraffin-embedded (FFPE) tissue samples (4% buffered formalin) from pre-therapeutic tumor biopsies and post-therapeutic resected specimens.

### Tumor DNA Preparation and Isolation

FFPE slides from pre-therapeutic and resected specimens were independently and blinded reevaluated by two experienced gastrointestinal pathologist (J.K., P.S.). Tumor regression grading (TRG) was assessed in percentage of regression in accordance with the Dworak Grading [19]. In discordant cases, slides were reassessed by both pathologists and a final decision was made. We added the respective tumor regression grades and relevant clinical data of the pretherapeutic biopsies for all patient samples in Table 1. Microdissection for tumor cell enrichment to achieve a content of 70–80% was performed manually. The technique was performed as recently reported by Hunt and colleagues [20]. Briefly, FFPE tissue slices were deparaffinized and stained with Haematoxylin. Representative tumor areas were identified using a microscope at a 40 fold magnification. Dissection was performed using a pointed surgical blade. Tumor tissue was transferred to a tube and DNA extraction was performed subsequently by using the Qiagen AllPrep DNA/ RNA FFPE Kit (Qiagen, Hilden, Germany) according to the manufacturer`s instructions.

**Table 1:** Clinical Data with Tumor Regression Grading.

ID	Gender	uT	uN	cM	Tumor height (cm)	pT	pN	TRG (%)	TRG (Dworak)
1	m	3	1	0	5	3	1	60	3
2	m	3	1	0	3	3	1	35	2
3	f	3	1	0	12	3	1	20	1
4	m	3	0	0	4	3	0	15	1
5	m	3	0	0	7	3	0	70	3
6	f	3	0	0	1	3	0	60	3
7	m	3	1	0	9	3	1	40	2
8	m	3	0	0	5	3	0	30	2
9	f	3	0	0	7	3	0	40	2
10	m	3	1	0	8	3	0	20	1
11	m	3	0	0	12	3	0	40	2
12	m	3	1	0	4	3	1	90	3
13	m	3	1	0	4	3	1	70	3
14	f	3	1	0	1	3	1	55	3
15	m	2	1	0	3	3	0	20	1
16	m	3	0	0	8	3	1	10	1
17	f	3	1	0	9	3	1	70	3
18	f	3	0	0	8	3	0	40	2
19	m	3	1	0	8	3	1	70	3
20	m	3	1	0	6	3	1	40	2
21	m	3	0	0	11	3	0	20	1
22	m	4	1	0	8	3	1	45	2
23	m	2	1	0	5	2	0	40	2
24	m	3	1	0	11	3	1	80	3
25	m	3	1	0	7	3	1	45	2
26	f	3	1	0	11	3	0	40	2
27	m	3	0	0	10	3	0	70	3
28	m	3	1	0	4	3	1	70	3
29	m	3	0	0	8	3	0	50	3
30	m	3	0	0	7	3	0	80	3
31	m	3	1	0	8	3	0	45	2
32	m	3	1	0	8	3	1	50	3
33	f	3	1	0	4	3	1	70	3
34	f	3	1	0	8	3	1	45	2
35	m	3	1	0	10	3	1	80	3
36	f	3	1	0	5	3	1	70	3
37	m	3	1	1	8	3	1	70	3
38	m	3	1	1	9	3	1	45	2
39	m	3	1	0	6	3	1	70	3
40	f	3	1	1	5	3	1	90	3
41	f	3	1	0	5	3	1	35	2
42	f	3	1	0	3,5	3	1	70	3
43	f	3	1	0	9	3	1	95	3
44	f	3	1	0	11	3	1	30	2
45	m	3	1	0	10	3	0	10	1
46	m	3	0	0	1	3	0	70	3
47	m	3	0	0	3	3	0	50	3

Relevant clinical data and TRGs in % and TRG according to Dworak for the analyzed patients (m = male, f = female, Tumor height in cm with respect to the anocutaneous line, ultrasonographic/clinical (u/c) and pathological (p) TNM stage: T = tumor; N = lymph node, M = metastasis, TRG = Tumor Regression Grading).

### Mutation Analysis

#### Primer Extension Method–SNaPshot™ Assay

Primer extension method was used to analyze known hot-spot *KRAS* mutations in rectal cancer [21] as previously described [22]. Briefly, regions of hotspot mutations in codons 12, 13, 61 and 146 were amplified by multiplex PCR using Qiagen Multiplex Kit (Qiagen, Hilden, Germany) with constantly 20 ng input DNA. This total amount of starting DNA was retained also for sensitivity testing, by which DNA with single known *KRAS* mutations was diluted with DNA harboring wildtype configuration at the indicated *KRAS* loci. Therefore, eight admixtures with 100, 50, 40, 30, 20, 10, 1, and 0% of mutant-containing DNA per each of the four interrogated mutations were prepared prior to subjection to multiplex PCR. After shrimp alkaline phosphatase and Escherichia coli exonuclease I (USB, Staufen, Germany) treatment specific primers binding adjacent to the potential mutation sites were applied and elongated by fluorescence-labeled dideoxynucleotide using the SNaPshot™ Multiplex Kit (Applied Biosystems, Foster City, CA, USA). GeneScan™ 120 LIZ^®^ Size Standard was used as an internal DNA sizing ladder for capillary electrophoresis (3100 Genetic Analyzer, Aplied Biosystems, Foster City, CA, USA). Results were analyzed with GeneScan™ Analysis Version 3.5.1 (Applied Biosystems, Foster City, CA, USA). Primer Extension technique has previously been shown to be a valid technique to identify mutations or polymorphisms [23, 24].

In case of discrepant results for KRAS mutation testing we reevaluated the sensitivity with the therascreen® KRAS testing system.

#### Therascreen® RAS Test

*The KRAS* Pyro Kit 24, V1 (Qiagen, Hilden, Germany) (cover mutations in *KRAS* codons 12, 13, and 61 of the human *KRAS* gene) and the *RAS* Extension Pyro Kit 24, V1 (Qiagen, Hilden, Germany) (cover mutations in *KRAS* codons 59, 61, 117 and 146 of the human *KRAS* gene) have been conducted for the validation detection of mutations of the *KRAS* gene in genomic DNA of rectal cancer specimen. The PyroMark Q24 MDx platform platform has been used to run the Therascreen® assay with the Software Q24, Version 2.0.7 with following PlugIns, *KRAS* PlugIn v1.2.0 and *RAS* Extention PlugIn v.1.2.1.2.

We amplified regions of interest in the extracted DNA using primers in the *KRAS* Pyro assay (QIAGEN, Hilden, Germany). We subsequently immobilized, washed, and denatured the amplified products using the vacuum workstation and subjected those products to pyrosequencing using the PyroMark Q24 Pyrosequencer (QIAGEN, Hilden, Germany) to detect and quantify the *KRAS* mutations. Initial DNA input for each specimen was 100 ng.

#### Cell lines

The following human colorectal cancer cell lines harboring indicated, distinct *KRAS* mutation have been used for the experiments described in this manuscript: DLD1 (ATCC CCL-221) for G13D, SW1116 (ATCC CCL-233) for G12A, LS174T (ATCC CCL-188) for G12D, and SKCO1 (ATCC HTB-39) for G12V. As a negative control, wild-type KRAS genomic DNA was obtained from the human fibroblast cell line BJ (ATCC: CRL-2522). All cell lines have been purchased from ATCC (Manassas, Virginia, USA).

## Results

**Fig 1:**
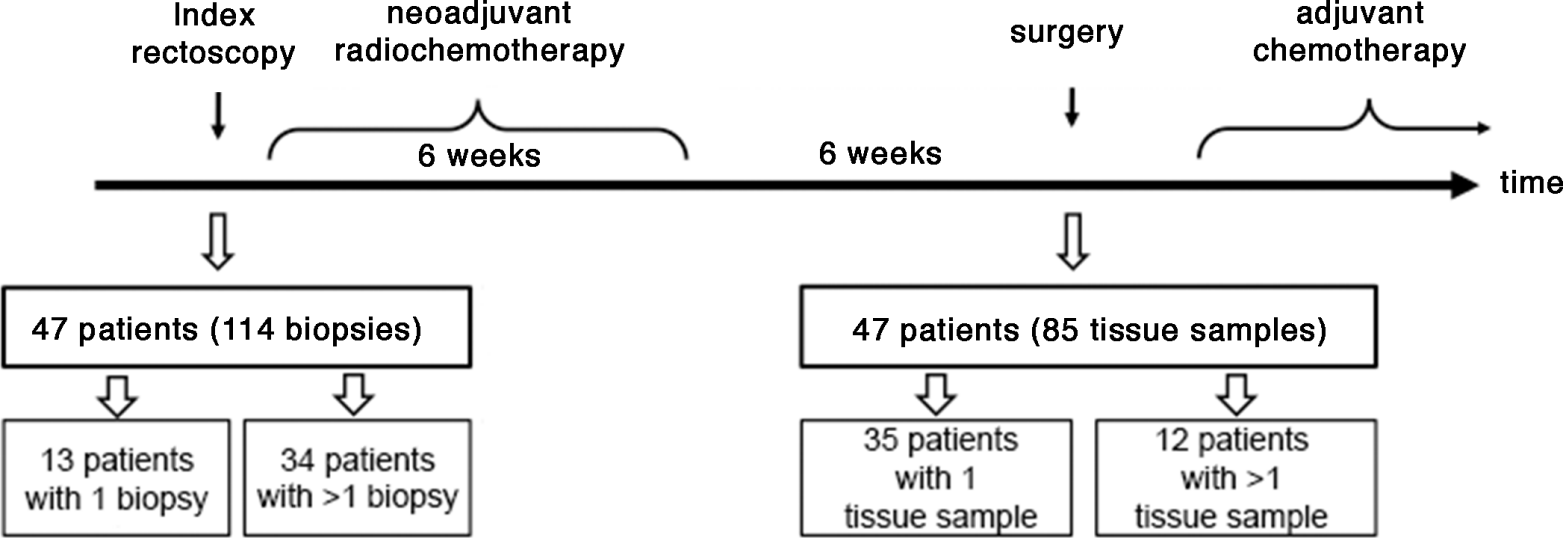
Analysis of pre- and post-therapeutic *KRAS* mutation status in 47 patients.

Treatment of patients suffering from locally advanced rectal cancer (UICC stage II and III) comprises neoadjuvant chemoradiotherapy, resection and adjuvant chemotherapy. In our study 32 (68.1%) male and 15 (31.9%) female patients received neoadjuvant CRT followed by surgical resection and adjuvant chemotherapy (Fig 1). Neoadjuvant CRT included irradiation of the presacral space with an overall dose of 50.4 Gy (single dose of 1.8 Gy) accompanied by either 5-fluorouracil (n = 24; 51.1%) or a combination of an intravenous infusion of oxaliplatin and a continuous infusion of 5-fluorouracil (n = 23; 48.9%). Within 4 to 6 weeks after completion of neoadjuvant CRT primary tumor resection was carried out, including complete mesorectal excision followed by adjuvant chemotherapy. *KRAS* mutation status was determined PCR-based SNaPshot™ technique from biopsies obtained by index rectoscopy and from resected samples as displayed in Fig 1.

### Primer Extension Method Assay Accuracy

**Fig 2:**
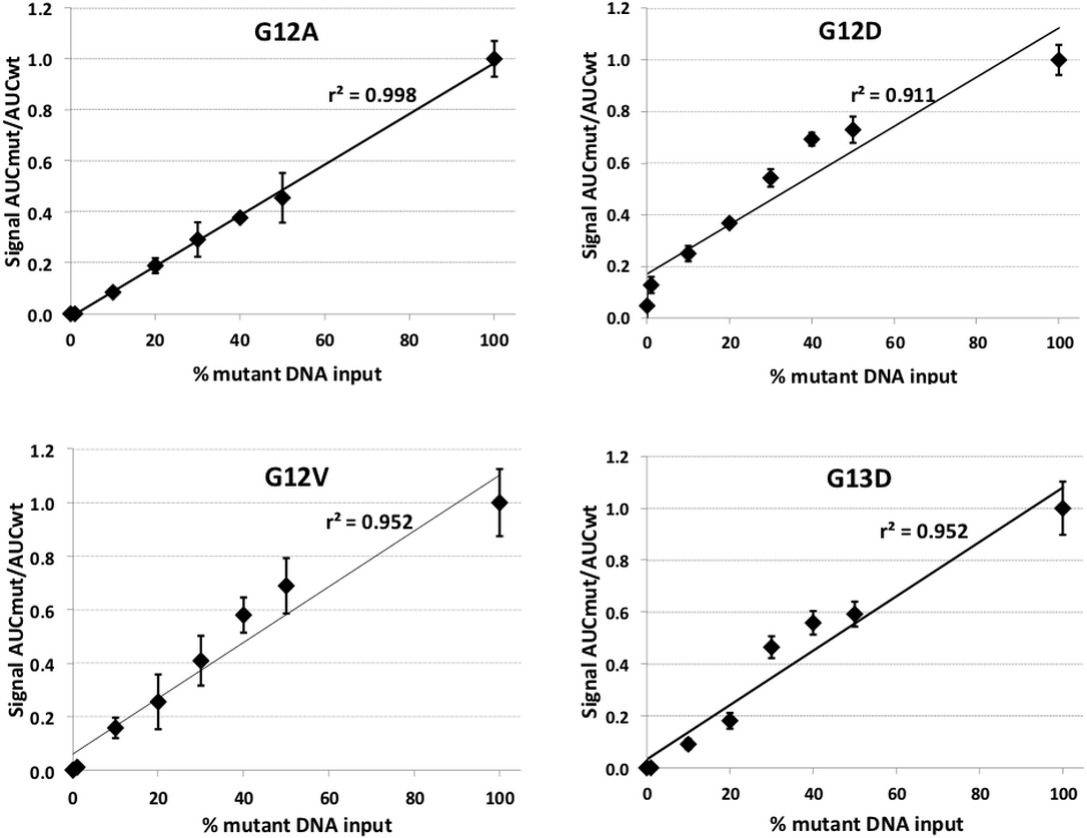
Sensitivity testing of the SNaPshot assay for G12A (using cell line SW1116), G12D (LS174T), G12V (SKCO1), and G13D (DLD1).

One of the major pitfalls in PCR-based detection of genomic mutation represents the sensitivity detection limit and the quality of primers used in the amplification step. In order to evaluate our primer system we used human colorectal cancer cell lines harboring distinct genomic *KRAS* mutations. To assess the detection limit of the primer extension assay we simulated possible contamination of tumor cells with fibroblasts. Precisely, we used four established cell lines as a model for one of the considered KRAS mutations: DLD1 for G13D, SW1116 for G12A, LS174T for G12D, and SKCO1 for G12V. For wild-type *KRAS* status genomic the human fibroblast cell line BJ was employed. Genomic DNA of each of the four tumor cell lines was serially diluted with that of the BJ line and subjected to amplification by PCR followed by SNaPshot™ assay to. evaluate sensitivity of this procedure. Therefore, we determined the signal strength, i.e. area under the curves (AUC), of peaks representing either wild-type or mutant *KRAS* allele in the prepared serial dilutions of positive and negative control and calculated a pair-wise ratio of corresponding peaks (Fig 2).

For all *KRAS* mutation variants, we could demonstrate that the applied SNaPshot™ technique represents a reliable test assay. Specific *KRAS* mutations G13D and G12A could be reliably detected up to a contaminating dilution with 90% fibroblast DNA, that of G12D even up to 99% and that of G12V up to 80% demonstrating high sensitivity and clearly suggesting this method being able to detect all considered *KRAS* mutations in the microdissected biopsies and resected specimen. Furthermore, in all test samples we correctly obtained the expected *KRAS* mutation variant defined by the used cancer cell line.

In each case, the signal intensity represented as the AUC (area under the curve) of the peak detected in the electropherogram for the mutant (AUCmut) in relation to the wildtype (AUCwt) allele was calculated. This ratio was each referred to the sample containing 100% input DNA of the cancer cell line with the respective mutation (set to 1.0). A regression line was calculated with the coefficient of determination (r^2^) indicated. Each series was assessed three times independently with the standard deviation for each dilution denoted as error bars.

### *KRAS* status between pre-therapeutic biopsies *versus* corresponding post-therapeutic resected specimen

**Fig 3:**
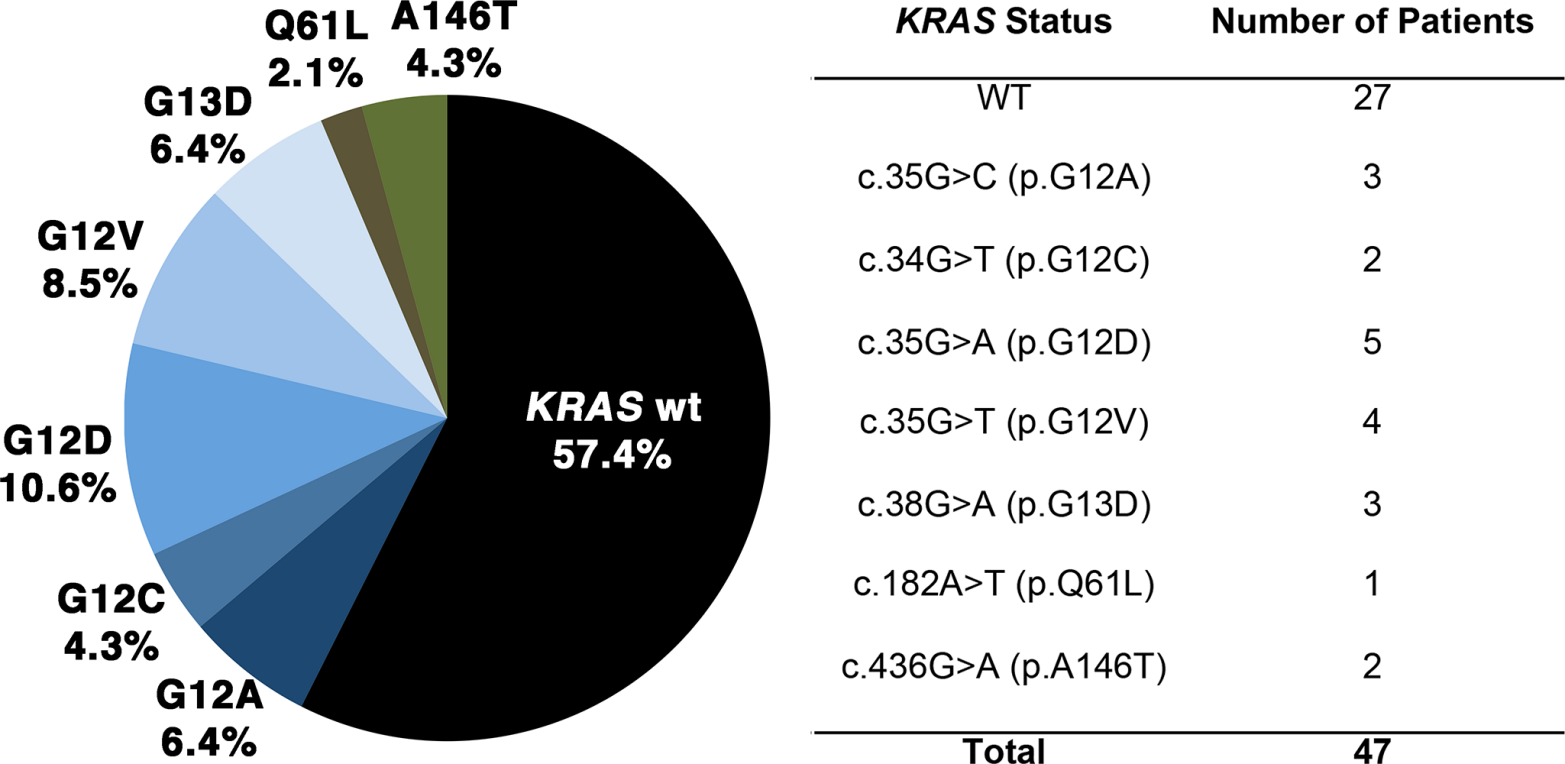
Distribution of *KRAS* mutation- and wild-type status in pre-therapeutic biopsies.

The *KRAS* mutation status of biopsies obtained at the index rectoscopy and corresponding resected specimens after chemoradiotherapy and subsequent resection was studied in 47 patients (Fig 1). In 20 out of these 47 patients (42.6%) we detected a mutation in either exon 2, 3, or 4 (Fig 3). The majority of these mutations (12/20) affected codon 35. Discrepancy of the *KRAS* mutation status in samples obtained at the index rectoscopy and in samples from the corresponding patients after CRT were found in six patients (12.8%) using the SNaPshot™ assay. All of them revealed a *KRAS* mutation in the pre-therapeutic biopsy but were determined to be wild-type if analysis was performed on a representative tissue block of the resected specimen.

The discrepant probes were reevaluated with an additional test system using the Therascreen® KRAS assay. Using this additional test assay, four out of these six samples revealed a concordant KRAS mutation in the samples obtained at the index rectoscopy and in the matched resected specimen. The remaining two cases could not have been reassessed since no DNA was available for reevaluation (Table 2). Additionally, the Therascreen® KRAS assay revealed the same KRAS mutation in samples from index rectoscopy and surgical resection which let us suggest these results being reliable.

**Table 2:** Determination of *KRAS* status in samples with discrepancy between samples from index rectoscopy and surgical resected specimen by using different assay systems (NA = not applicable).

PatientID	*KRAS* mutation status SNaPshot™ assay		*KRAS* mutation status Therascreen® assay	
	Index rectoscopy	Resected sample	Index rectoscopy	Resected sample
36	A146T	wild-type	A146T	A146T
41	G13D	wild-type	G13D	G13D
44	G12V	wild-type	G12V	G12V
47	G12V	wild-type	G12V	G12V
39	G12V	wild-type	NA	NA
26	G12A	wild-type	NA	NA

### Intratumoral heterogeneity within biopsies from index rectoscopy and surgically resected specimens

**Fig 4:**
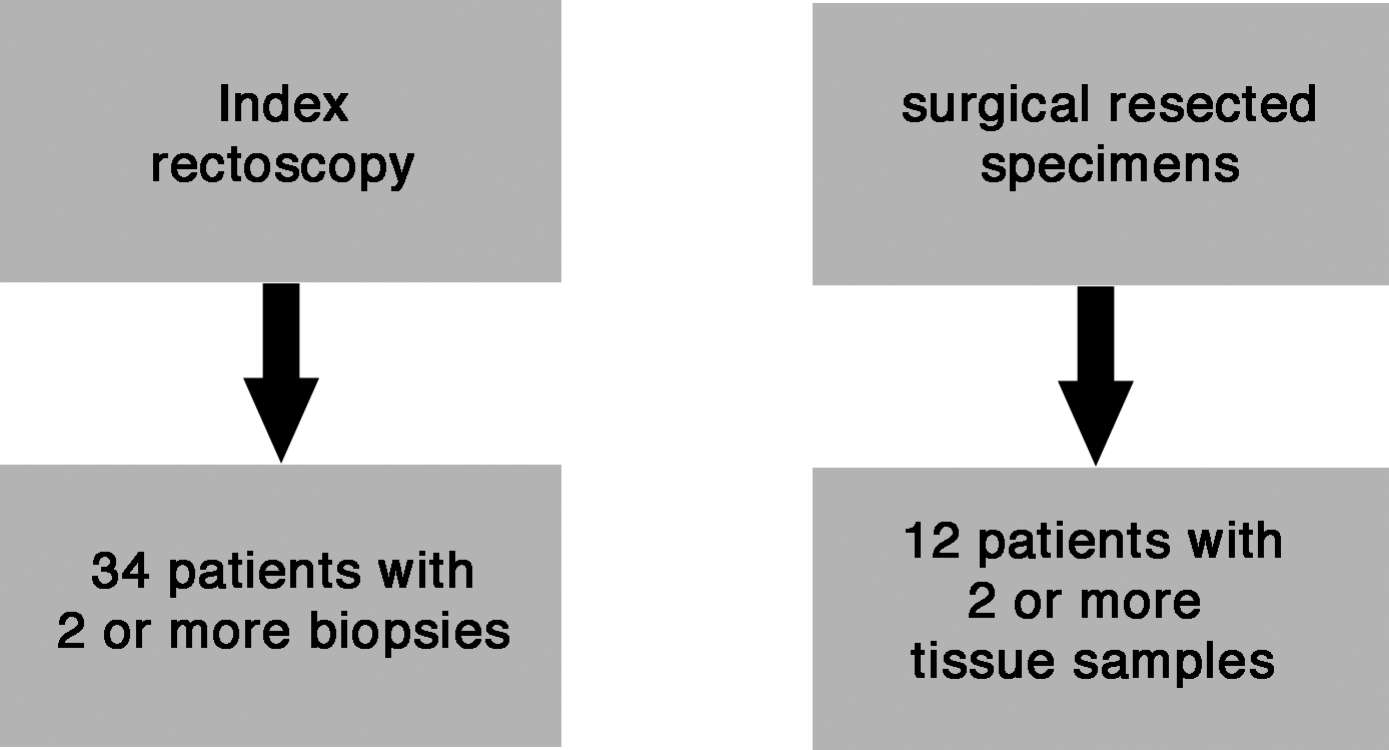
Figure showing tumor samples used for analysis for intratumoral heterogeneity within biopsies from index rectoscopy and surgically resected specimens.

A potential problem in the determination of the *KRAS* mutation status by collecting a single biopsy represents the sampling error. This pitfall in diagnosis is based on the assumption that distinct clones of tumor cells exists in solid tumors harboring potential heterogeneity in *KRAS* mutations. To assess the role of intratumoral heterogeneity between several biopsies within the tumor, *KRAS* mutation status was assessed in multiple biopsies obtained at the index rectoscopy as well as in tumor blocks from the surgically resected specimen. For this analysis only cases with more than one tissue sample (biopsies from index rectoscopy: range 2–5 biopsies per patient, surgically resected specimens: 3–5 tissue blocks per patient) were included (Fig 4).

On average, we obtained three biopsies at the index rectoscopy and subjected these samples for *KRAS* analysis. Only in a single patient we found discordant results for *KRAS* testing in two different biopsies from the same tumor analyzed by the SNaPshot™ assay. One sample revealed a G12V mutation and the other *KRAS* wild-tpye status. These two discordant DNA samples were referred to therascreen® KRAS testing. Unfortunately, the analysis failed since no peaks were detectable. For re-testing no sufficient DNA amount was available.

Due to the high tumor regression induced by preoperative CRT the intratumoral heterogeneity in the surgically resected specimen could only be assessed in a subset of 12 patients, for whom more than one tumor-bearing block was available (average of 4.2 blocks/patient). In 6 out of 12 patients we found a homogenous *KRAS* status comparing at least two blocks using the SNaPshot™ assay. In the other 6 patients we detected discrepant results for the *KRAS* mutation status (Table 3). After reevaluation with the therascreen® *KRAS* test we could again confirm exactly that *KRAS* mutations, which were ascertained in the prior biopsy by the SNaPshot™ assay. Upon correction of the discordant data all tumor blocks showed a homogenous *KRAS* status.

**Table 3:** Assessment of intratumoral heterogeneity in resected specimens.

Patient ID	Biopsy from index rectoscopy	Resected Specimen	SNaPshot™assay	Therascreen® assay
6	G12D	Block 1	WT	G12D
		Block 2	G12D	G12D
		Block 3	G12D	G12D
8	G13D	Block 1	WT	G13D
		Block 2	WT	G13D
		Block 3	WT	G13D
		Block 4	G13D	G13D
13	G12V	Block 1	WT	G12V
		Block 2	WT	G12V
		Block 3	WT	G12V
		Block 4	G12V	G12V
22	G12D	Block 1	WT	G12D
		Block 2	WT	G12D
		Block 3	WT	G12D
		Block 4	WT	G12D
		Block 5	G12D	G12D
24	A146T	Block 1	WT	A146T
		Block 2	A146T	A146T
		Block 3	WT	.A146T
33	G12D	Block 1	G12D	G12D
		Block 2	G12D	G12D
		Block 3	WT	G12D
		Block 4	WT	G12D

Distribution of *KRAS* mutation status in different resected specimen tissue blocks, SNaPshot™ assay vs. Therascreen® *KRAS* test results. The Therascreen® *KRAS* test results show a homogenous mutation status in all evaluated FFPE tissue blocks.

## Discussion

As the main result of this study in rectal cancer there seems to be no apparent heterogeneity in the *KRAS* mutation status. This applies for specimens taken from tumors at the same occasion and for samples ascertained prior and upon CRT. This analysis is based on a high number of pre- and posttherapeutic samples and therefore represents a rare data set. Given a sensitive detection method in relation to the amounts of viable tumor cells, determination of the *KRAS* mutation status can be conducted reliably in tumor samples of the pre-therapeutic biopsy obtained at the index rectoscopy as well in the resected specimens after neoadjuvant chemoradiotherapy. With respect to the pre-therapeutic specimens naive for radio- and chemotherapy the SNaPshot™ assay appears to provide a reliable detection tool, in particular if multiple biopsies are assayed to minimize the risk of false-negative results. The overall numbers of *KRAS*-mutated tumors in the pre-therapeutic biopsies of 43% (20/47) matched pretty much current literature data in this issue. Moreover, the distribution of the single mutations observed was also much in line with that so far reported [25, 26]. This makes us confident that the SNaPshot™ assay produces reliable results in the pre-therapeutic samples, i.e. when sufficient numbers of viable tumor cells are present. Regarding specimens obtained upon chemoradiotherapy, our study suggests that a particular sensitive method like the FDA-approved Pyro Therascreen® kit should be preferred.

Our results support findings by Ondrejka et al. [14] who did not find any discrepancy when the *KRAS* status in 17 patients with rectal cancer was assessed before and after neoadjuvant CRT. First results of next generation sequencing and quantitative analyses show *KRAS* mutations in the vast majority of neoplastic cells [27]. In a study recently published by Demes et al. [15] in two out of 25 patients a discrepant *KRAS* status in samples obtained before and after therapy was detected. As the two techniques applied (sequencing and SNaPshot™) are much related it can be hypothesized that the sensitivity in that study was comparable to the SNaPshot™ technique of our study and may have failed to identify mutations in highly degraded samples. This assumption is consistent with Boissière-Michot et al. [16] who recently pointed out that especially in rectal cancer after CRT false negative detection of the *KRAS* mutation status represents a relevant problem possibly leading to serious malpractice. This refers to technical issues as a second important result of this study.

Whereas the SNaPshot™ assay appears to have a good sensitivity to detect *KRAS* mutations in viable tissues (i.e. pre-therapeutic biopsies) this technique does not sufficiently detect mutated loci upon neoadjuvant CRT. The most plausible explanation is a massive therapy-provoked decay of tumor cells. Inflammatory and stromal reactions result in a substantial increase of non-mutated cells in the tumor area thus “diluting” the remaining malignant cells. However, the application of a highly sensitive technique for mutation analysis revealed the mistakenly observed changes. This is in line with a study by Gonzalez de Castro D et al. having compared sensitivity of Sanger sequencing, on which the SNaPshot™ technique is actually based, with cobas, therascreen and massive parallel pyrosequencing [28] In case of low fractions of mutant DNA the Sanger sequencing was less sensitive than the other investigated methods.

Boissière-Michot et al. [16] suggested that higher sensitivity can be achieved by laser microdissection and the use of the therascreen® assay. In the present study we can show that manual microdissection appears to be sufficient to gather adequate amounts of tumor cells for DNA analysis. This may be relevant for clinical practice especially in less specialized centers where laser microdissection of tumor tissue is not frequently available.

As pointed out data on *KRAS* status testing are still rare to assess the true rate of discrepant cases. By adding the herein largest patient cohort and analyzing the *KRAS* status in different pre-therapeutic biopsies from the same tumor we can add additional confidence to the clinical practice of *KRAS* testing in rectal cancer. Furthermore, we agree with Boissière-Michot et al. [16] that the type of technique used for testing should be performed according to the available tumor tissue and financial resources are of importance if initial tumor tissue is of minor quantity or quality. This is of importance as patients receiving anti-EGFR therapy having a *KRAS* mutation suffer from unnecessary side effects [29].

## Conclusions

In summary, our results indicate the reliable assessment of *KRAS* mutation status for therapeutic decisions in pre-therapeutic biopsies as well as in post-therapeutic residual tumor tissue. Discordance may be a very rare event and is practicably ignorable. The SNaPshot™ assay can be used cost effectively for mutational analysis of tumor samples with a high tumor cell content, whereas more sensitive and expensive tests should be reserved for inconclusive cases and for samples with a low amount of tumor cells such as those after CRT.
